# Ultrasound-assisted dispersive micro-solid phase extraction using molybdenum disulfide supported on reduced graphene oxide for energy dispersive X-ray fluorescence spectrometric determination of chromium species in water

**DOI:** 10.1007/s00604-020-04476-4

**Published:** 2020-09-02

**Authors:** Katarzyna Pytlakowska, Karina Kocot, Michał Pilch, Maciej Zubko

**Affiliations:** 1 Institute of Chemistry, University of Silesia, ul. Szkolna 9, 40-006 Katowice, Poland; 2 Institute of Physics, University of Silesia, 75 Pułku Piechoty 1a, 41-500 Chorzów, Poland; 3 Institute of Materials Science, University of Silesia, 75 Pułku Piechoty 1a, 41-500 Chorzów, Poland

**Keywords:** Preconcentration, Hexavalent chromium determination, Chromium speciation, EDXRF, Environmental samples

## Abstract

Molybdenum disulfide (MoS_2_) was supported on graphene oxide (GO) by hydrothermal method. The resulting nanocomposite (MoS_2_-rGO) was characterized by X-ray photoelectron spectroscopy, scanning electron microscopy, and transmission electron microscopy. The experiments show that at pH 2, MoS_2_-rGO has a great affinity for adsorption of hexavalent chromium ions while Cr(III) ions remain in aqueous sample. In the adsorption process, the dominant role plays chemisorption. The determined adsorption capacity is 583.5 mg g^−1^. Parameters affecting the extraction process, namely sample pH, sample volume, contact time, and matrix ions, were investigated by sequential batch tests. Under optimal conditions (pH 2, sample volume 50 mL, sonication time 10 min, adsorbent mass 1 mg), the calibration curve covers the 1–200 ng mL^−1^ range with a correlation coefficient (*R*^2^) of 0.998. The recovery of the method is 97 ± 3%. Other data of merit include a relative standard deviation of < 3.5%, enrichment factor of 3350, and detection limit of 0.050 ng mL^−1^. The accuracy of the method was confirmed by analysis of the reference materials QC1453 (chromium VI in drinking water) and QC3015 (chromium VI in seawater). The method was successfully applied to chromium speciation in water samples, including high salinity ones. The concentration of Cr(III) was calculated as the difference between the total concentration of chromium (after oxidation of Cr(III) to Cr(VI) with potassium permanganate) and the initial Cr(VI) content.

Graphical abstract

**Electronic supplementary material:**

The online version of this article (10.1007/s00604-020-04476-4) contains supplementary material, which is available to authorized users.

**Figure:**
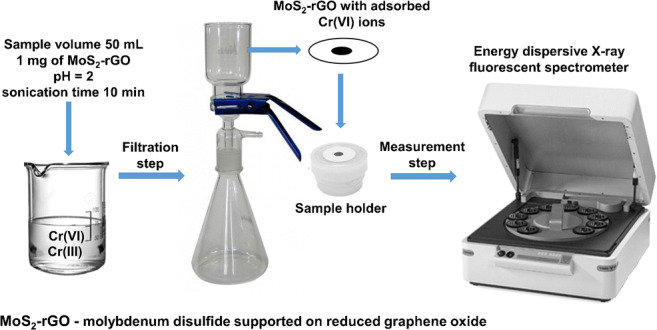
Schematic presentation of a method for determination of chromium species by energy dispersive X-ray fluorescence spectrometry after preconcentration on molybdenum disulfide supported on reduced graphene oxide.

## Introduction

Determination of chromium species in waters is of prime interest due to their contrasting bioavailability and physiological properties. According to the World Health Organization (WHO) [1] and the United States Environmental Protection Agency (EPA) [2], the permissible level of total chromium content in drinking water is 50 ng mL^−1^ and 100 ng mL^−1^, respectively. Most analytical techniques including inductively coupled plasma atomic emission spectrometry (ICP-OES), inductively coupled plasma mass spectrometry (ICP-MS), flame and electrothermal atomic absorption spectrometry (FAAS and ET-AAS), energy dispersive X-ray fluorescence spectrometry (EDXRF), or total reflection X-ray fluorescence spectrometry (TXRF) enable determining total concentration of chromium in environmental samples. Thus, isolation and/or preconcentration step is necessary to determine chromium species.

According to the literature, there are three main procedures for chromium speciation: (i) selective preconcentration of Cr(III) or Cr(VI) and further oxidation or reduction determining its total concentration, (ii) preconcentration of both chromium states at different pH values and subsequent elution with different solvents, or (iii) preconcentration of both forms of chromium at the same sample pH and subsequent elution with different solvents. The first approach is the most popular in chromium speciation.

Among a vast array of sample pretreatment methods used for chromium speciation, solid phase extraction (SPE) and dispersive solid phase extraction (DSPE) have been the most often utilized. Their widespread use results from simplicity, relatively low costs, the ability to process large volume samples, fast and easy phase separation, minimized solvent consumption, possibility of obtaining high enrichment factors, easy coupling with different analytical techniques, and many existing adsorbents [3]. However, classical adsorbents, such as activated carbon [4], modified silica gel [5], chelating resins [6, 7], and Amberlite XAD-4 resin series [8, 9], have been gradually replaced by nanosized adsorbents due to their unique large surface area, high adsorption capacity, and chemical stability. For preconcentration of chromium species from environmental samples, various types of nanomaterials including oxidized multiwalled nanotubes (MWCNTs) [10], MWCNTs and graphene oxide (GO) modified with Aliquat 336 [11, 12], MWCNTs modified with 3-(2-aminoethylamino) propyltrimethoxysilane (AAPTS-MWCNTs) [13], GO decorated with triethylenetetramine-modified magnetite (mf-GO) [14], GO functionalized with 3-(2-aminoethylamino) propyltrimethoxysilane (GO-1N) [15], and molybdenum disulfide (MoS_2_) [16] were used. MoS_2_ is the best-known and widely explored representative of the transition metal dichalkogen (TMDs) group. Its crystals consist of hexagonal molybdenum layers disposed between two sulfur layers by covalent bonds. These bonds are responsible for excellent mechanical strength and thermal stability up to 1090 °C in an inert atmosphere environment. Weak van der Waals bonds allow forming thin MoS_2_ layers of a single or a multi-layer thickness. The presence of a large amount of sulfur atoms on surfaces and edges which act as a soft Lewis base makes MoS_2_ excellent adsorbent for the removal of heavy metal ions that behave like soft Lewis acids. Its defect-rich structure formed during synthesis together with enlarge interlayer spacing between the three atom layers (S-Mo-S) allows penetrating small molecules or ions into MoS_2_ layers. It results in increase of both its adsorption capacity and possibility of surface modification [17]. For chromium preconcentration, different composites of MoS_2_ including magnetic nanoparticles (Fe_3_O_4_NPs) decorated with MoS_2_ (MoS_2_@Fe_3_O_4_) [18], sodium dodecyl sulfate (SDS)-intercalated MoS_2_ (SDS-MoS_2_) [19], MoS_2_ coated Mg/Al layered double hydroxide composite (LDHs@MoS_2_) [20], polyvinylpyrrolidone (PVP), and polyacrylamide (PAM) intercalated MoS_2_ composites [21] were successfully applied.

Membrane-based systems for chromium preconcentration were also proposed [22, 23]. Their combination with XRF techniques simplifies procedures by elimination of elution of target ions and centrifugation or filtration step. As a result, the risk of the analyte loss or sample contamination is minimized. The main drawback of such approach is the time required to establish the equilibrium state that is much longer than in the case of DSPE.

Herein, a method for chromium speciation in waters by energy dispersive X-ray fluorescence spectrometry (EDXRF) after preconcentration on molybdenum disulfide supported on reduced graphene oxide (MoS_2_-rGO) is described. The method is based on a selective adsorption of hexavalent chromium on the MoS_2_-rGO surface. The concentration of Cr(III) is calculated as the difference between the total concentration of chromium (after oxidation of Cr(III) to Cr(VI) with potassium permanganate) and the initial Cr(VI) content. Considering that the adsorption of Cr(VI) occurs at acidic conditions, the method is resistant to high concentration of other coexisting anions, alkali, and alkaline earth cations usually present in water samples and for that reason can be used for sensitive hexavalent chromium determination and speciation in high salinity samples. The synthesis of MoS_2_-rGO, its structural characterization, the adsorption process optimization, and validation of the method were carefully studied. The research broadens the scope of MoS_2_-rGO application since its first successful use for Pb(II) and Ni(II) preconcentration from urine, saliva, and water samples prior to AAS determination [24].

## Experimental

### Reagents and solutions

Stock solutions of Cr(III), Cr(VI), Se(IV), Se(VI), As(III), and As(V) (1 mg mL^−1^), tannic acid, nitric acid (65%, Suprapur®), and ammonium hydroxide solution (25%, Suprapur®) were purchased from Merck (Darmstadt, Germany, www.merckgroup.com). Salts used for the interferences studies, potassium permanganate, sulfuric acid, sodium molybdate dihydrate, hydrochloride acid, and ethanol were purchased from POCh (Gliwice, Poland, www.poch.com.pl). Graphite powder (325 mesh) was purchased from Alfa Aesar (Karlsruhe, Germany, www.alfa.com). L-cysteine, humic acid, and certified materials: chromium VI in drinking water (QC1453) and chromium VI in seawater (QC3015), were purchased from Sigma-Aldrich (Laramie, Wyoming, USA, www.sigmaaldrich.com). High-purity water obtained from a Milli-Q system (Millipore, Molsheim, France, www.merckmillipore.com) was applied in the whole studies.

### Instruments

X-ray photoelectron spectroscopy measurements were performed with a PHI 5600 Physical Electronic Spectrometer (www.phi.com) with the use of monochromated Al Kα radiation, at the ultra-high vacuum pressure of 5 × 10^−10^ mbar, 15 kV, and 20 mA. The energy resolution was 0.1 eV. All photoelectron spectra were calibrated against the peaks of Au 4f_7/2_ at 83.98 eV, Ag 3d_5/2_at 368.27 eV, and Cu 2p_3/2_ at 932.67 eV of binding energy. The analysis of the surfaces of the powdered material was carried out at take-off angle 45°. The JEOL-5410 scanning electron microscope (SEM) and JEOL JEM 3010 transmission electron microscope (TEM) (www.jeol.co.jp/en) were used to the MoS_2_-rGO surface observations. Determination of Cr(VI) and chromium speciation was conducted on an Epsilon 3 EDXRF spectrometer (PANalytical, Almelo, The Netherlands, www.panalytical.com). A spectrometer equipped with a 200-μm Al primary beam filter worked at 30 kV and 0.300 mA, in atmospheric conditions. A counting time of 300 s was used in all EDXRF measurements. Adsorption capacity of MoS_2_-rGO and recovery research were performed using a SpecroFMS16a spectrometer with excitation in the ICP plasma (Spectro Analytical Instruments, www.spectro.com). A spectrometer worked at the following conditions: plasma power 1.4 kW, coolant gas Ar, 12 L min^−1^, auxiliary gas Ar, 1 L min^−1^, nebulizer gas Ar, 1 L min^−1^, nebulizer pressure 3.2 bar, nebulizer cross-flow type, sample uptake rate 2 mL min^−1^, wavelength 284.3 nm.

### Synthesis of MoS_2_-rGO

**Fig. 1:**
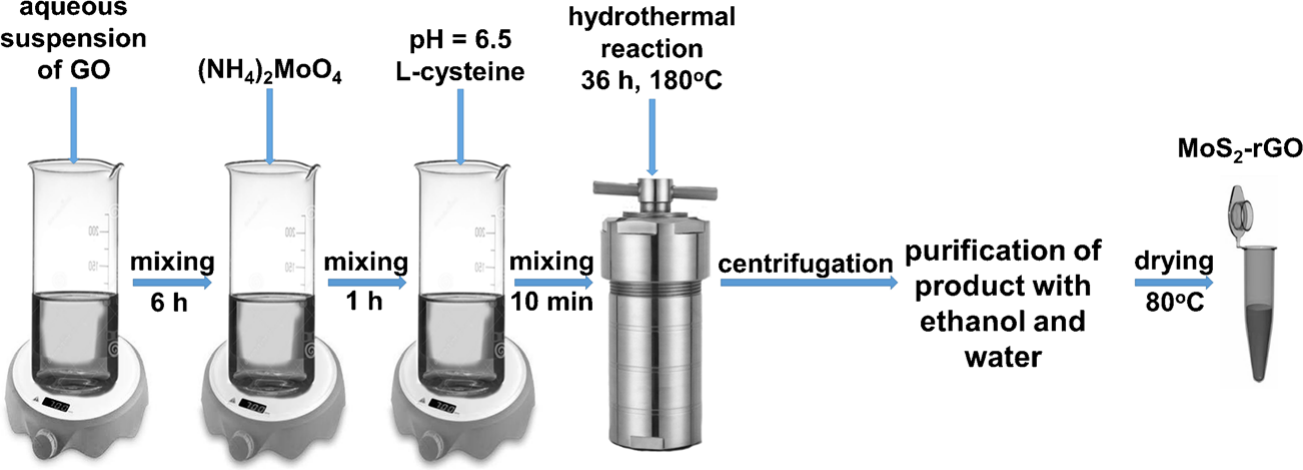
Scheme of synthesis of MoS_2_-rGO nanocomposite

Graphene oxide applied to MoS_2_-rGO synthesis was prepared by the modified Hummers’ method [25]. Synthesis of MoS_2_-rGO nanoparticles was conducted according to the literature data [24]. Succinctly summarizing, the 25 mL of aqueous suspension containing 0.1 g of GO was stirred at 900 rpm for 6 h at room temperature. Subsequently, 0.5 g of Na_2_MoO_4_·2H_2_O was added to the mixture and stirring was continued for the next hour. Then, suspension pH was adjusted to 6.5, and the solution containing 1 g of L-cysteine in 50 mL of water was added. The resulting mixture was transferred into a 100-mL Teflon-lined stainless steel autoclave and heated at 180 °C for 36 h in a laboratory dryer. The synthesized MoS_2_-rGO in the form of black precipitate was separated by centrifugation, washed 10 times with ethanol and water in order to remove an excess of L-cysteine and Na_2_MoO_4_·2H_2_O, and then dried at 80 °C. The scheme of MoS_2_-rGO synthesis is shown in Fig. 1.

### Batch adsorption studies for Cr(VI)

To 25 mL in volume aqueous samples containing appropriate amount of Cr(VI) ions, 1 mg of MoS_2_-rGO was added, and after pH adjustment to 2, the resulting suspensions were stirred at 900 rpm for 3 h at room temperature. Next, the aqueous suspensions were filtered with 0.22-μm membrane filters, and filtrates were collected in test tubes for further ICP-OES measurements. The metal ion concentration adsorbed on the MoS_2_-rGO surface (mg g^−1^) was calculated using the following equation: q_e_ = [(C_o_-C_e_)·V]/m_adsorbent_, where C_o_ is the initial concentration of Cr(VI) in aqueous solution (mg L^−1^), C_e_ is the equilibrium concentration (mg L^−1^), V is a suspension volume, and m_adsorbent_ is MoS_2_-rGO mass (mg).

### Ultrasound-assisted dispersive micro-solid phase extraction for Cr(VI)

One milligram of MoS_2_-rGO was added to 50 mL of aqueous sample containing different concentrations of Cr(VI) ions. Then, the pH was adjusted to 2 with 0.1 mol L^−1^ HNO_3_, and subsequently, the suspension was sonicated for 10 min. In the next step, the sample was filtered through a membrane filter (0.22 μm) under reduced pressure with the use of a customized filtration assembly of 5-mm diameter. The filter covered with MoS_2_-rGO and adsorbed Cr(VI) ions was dried under air conditions prior to EDXRF measurement. The blank sample was prepared in the same way as described above, but high-purity water was used instead of the examined sample.

### Chromium speciation

In order to carry out the speciation analysis, two aliquots of a sample are required. The first aliquot is analyzed for the Cr(VI) content, while the second one for the total chromium concentration determined after the oxidation of Cr(III) ions to Cr(VI) with potassium permanganate [26]. The procedure was as follows: 4–5 drops of 0.02 mol L^−1^ KMnO_4_ were added to 50 mL of a sample at pH 2. Then, the solution was heated for 15 min under cover at 90–95 °C. After cooling the sample, preconcentration of Cr(VI) was performed according to the procedure described above. The Cr(III) amount was calculated as the difference between the total chromium and initial Cr(VI) content.

### Real samples and sample preparation

Lake, spring, and river waters, collected in the Upper Silesian region (Poland), were filtered through a 0.22-μm cellulose acetate membrane (Millipore), and after acidification with HNO_3_ stored without access of light at 4 °C. The artificial sea water was prepared by dissolving 21.03 g NaCl, 3.52 g Na_2_SO_4_, 0.61 g KCl, 0.088 g KBr, 0.034 g Na_2_B_4_O_7_ ∙ 10H_2_O, 9.50 g MgCl_2_ ∙ 6H_2_O, 1.32 g CaCl_2_ ∙ 2H_2_O, 0.02 g SrCl_2_ ∙ 6 H_2_O, and 0.02 g NaHCO_3_ in 1 L of high-purity water [27].

## Results and discussion

### Characterization of MoS_2_-rGO

**Fig. 2:**
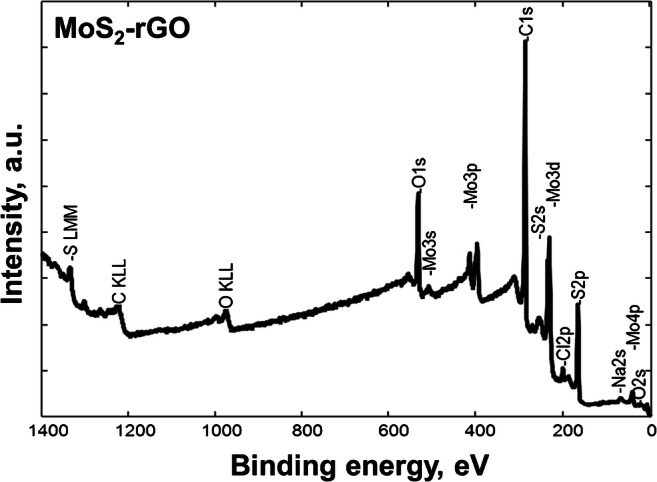
XPS survey spectrum of MoS_2_-rGO

The synthesized composite was characterized by XPS. Figure 2 shows the survey spectrum of MoS_2_-rGO consisted of carbon, oxygen, sulfur, and molybdenum states which is in accordance with its composition. The additional Na 2s and Cl 2p states are typical for human sweat.

**Fig. 3:**
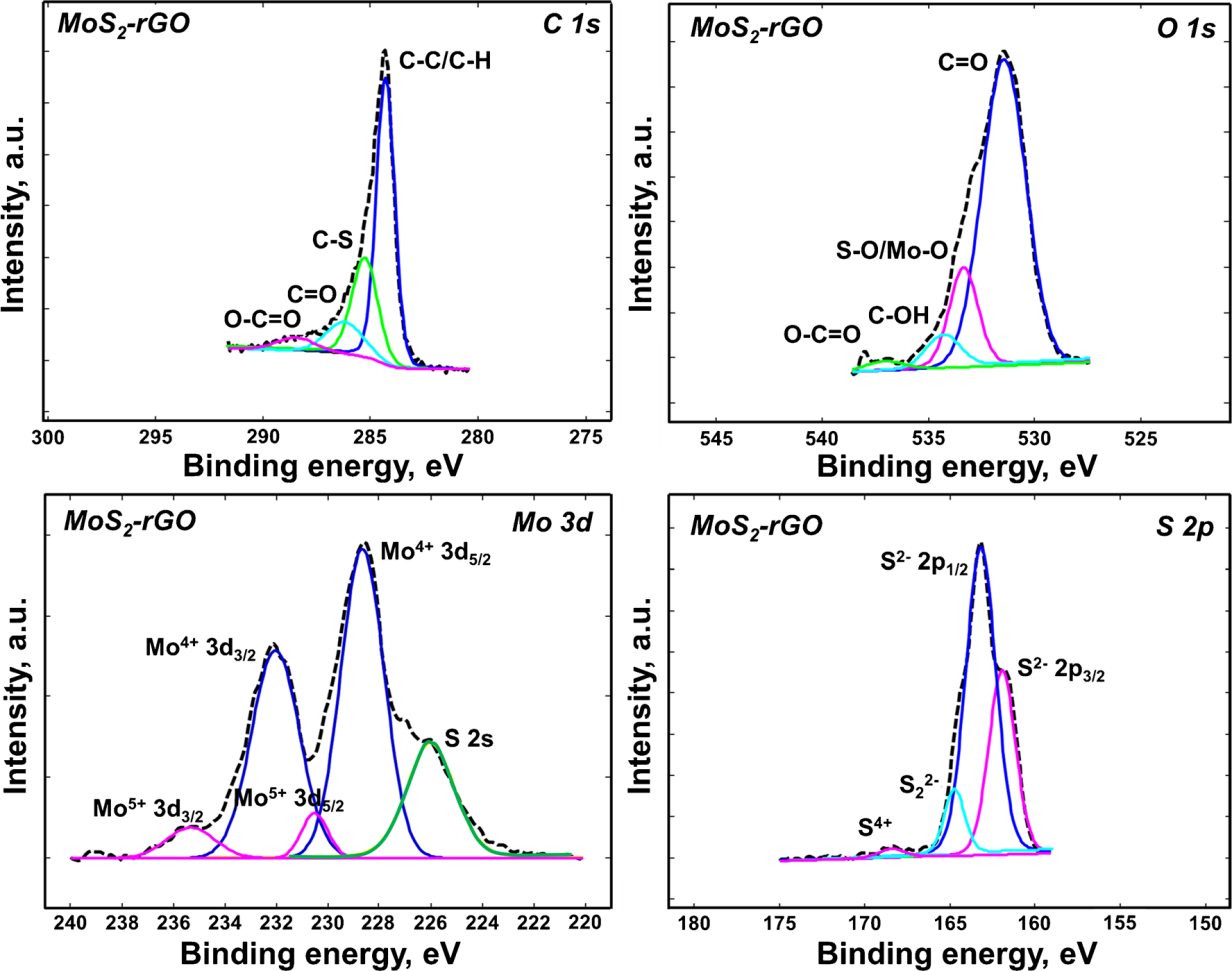
High-resolution C1s, Mo3d, S2p, and O1s spectra of MoS_2_-rGO

Figure 3 shows the high-resolution C1s, Mo3d, S2p, and O1s spectra of MoS_2_-rGO. The C1s spectrum was deconvoluted into four peaks at 284.5 (C-C), 285.6 (C-S), 286.2 (C=O), and 288.7 (O-C=O) eV. The Mo3d spectrum of MoS_2_-rGO shows not only two doublets Mo^4+^ (3d_5/2_ at 228.5 eV, 3d_3/2_ at 232.5 eV) and Mo^5+^ (3d_5/2_ at 230.5 eV, 3d_3/2_ at 235.2 eV) but also S2s state at 226 eV. The S2p of MoS_2_-rGO reveals four peaks at 162.2 eV(S^2−^ 2p_3/2_), 163.3 eV (S^2−^ 2p_1/2_), 164.7 eV (S_2_^2−^), and 168.5 eV (S^4+^). The O1s of MoS_2_-rGO reveals four peaks at 531.5, 533.4, 534.3, and 537 eV assigned to C=O, Mo-O/S-O, C-OH, and O-C=O. Those changes of shape and intensity at the maximum position of C1s peaks involve successful modification of the material. Mo3d, S2p, S2s, and also both C1s and O1s deconvoluted lines indicate the functionalization of GO. It can be seen that new peaks (C–S in carbon line and Mo-O/S-O in oxygen line) assigned to surface groups are present in the spectra. The location of main peaks on the deconvoluted XPS spectra is in a good agreement with those described in the literature data [28].

**Fig. 4:**
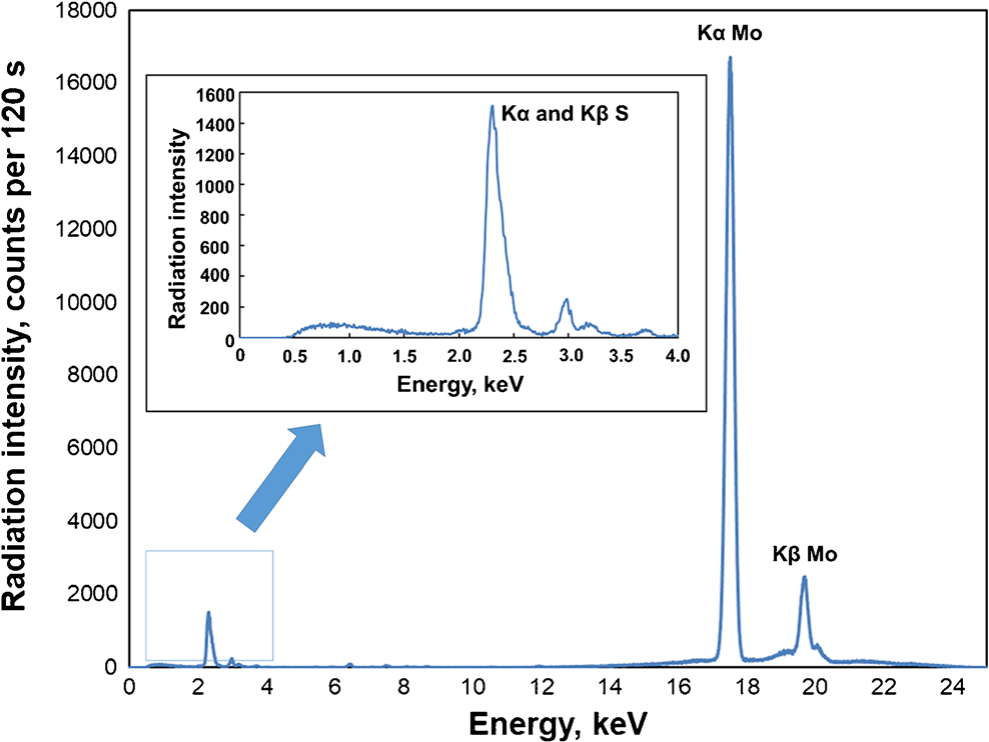
EDXRF spectrum of MoS_2_-rGO (Ag primary beam filter, 30 keV, 0.300 mA, counting time 120 s)

The modification of graphene oxide surface with molybdenum disulfide nanoparticles was also confirmed by the EDXRF technique. The presence of sulfur (overlapped Kα at 2.31 keV and Kβ at 2.46 keV) and molybdenum (Kα at 17.48 keV and Kβ at 19.61 keV) peaks in the recorded spectrum (Fig. 4) proves successful decoration of MoS_2_ on GO surface.

**Fig. 5:**
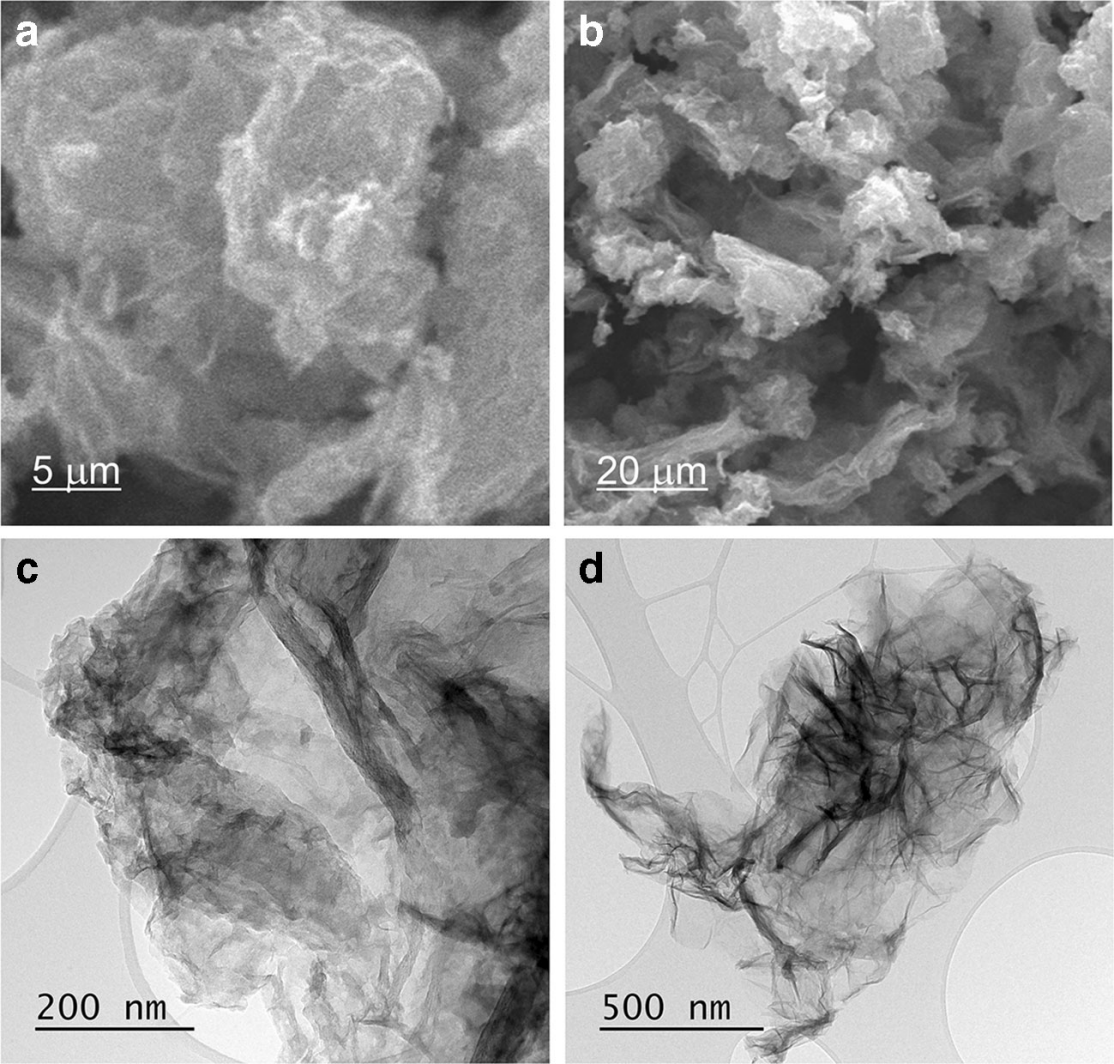
**a** and **b** SEM and **c** and **d** TEM images of MoS_2_-rGO surface at different magnifications

The SEM and TEM images of MoS_2_-rGO surface at different magnifications are presented in Fig. 5. The strongly undulating surface of the nanomaterial indicates a large surface area of a single flake which may result in a high extractive capacity.

### Optimization of ultrasound-assisted dispersive micro-solid phase extraction for Cr(VI)

The following parameters were optimized: (a) pH value; (b) sample volume; (c) contact time; (d) adsorbent mass. Respective text and figures on optimizations are given in the Electronic Supporting Material. In short, the following experimental conditions were found to give best results: (a) pH value, 2; (b) sample volume, 50 mL; (c) contact time, 10 min; (d) adsorbent mass, 1 mg.

### Adsorption isotherms for Cr(VI)

In order to clarify the adsorption mechanism of Cr(VI) ions on MoS_2_-rGO surface, the Langmuir [29] and Freundlich [30] isotherm models were employed. The isotherms were computed using the following equations:

$$ {\mathrm{q}}_{\mathrm{e}}=\frac{{\mathrm{q}}_{\mathrm{max}}{\mathrm{K}}_{\mathrm{L}}{\mathrm{C}}_{\mathrm{e}}}{1+{\mathrm{K}}_{\mathrm{L}}{\mathrm{C}}_{\mathrm{e}}}\; and\;{\mathrm{q}}_{\mathrm{e}}={\mathrm{K}}_{\mathrm{F}}{\mathrm{C}}_{\mathrm{e}}^{1/\mathrm{n}} $$

where q_max_ is the maximum amount of Cr(VI) ions adsorbed on 1 mg of MoS_2_-rGO surface to form a single layer coverage at highest equilibrium ion concentration (mg g^−1^), K_L_ is the adsorption enthalpy (L mg^−1^), K_F_ (mg^1-n^ L^n^ g^−1^) and *n* are Freundlich constants related to the adsorption facility and adsorption intensity, respectively.

The course of adsorption isotherms as well as parameters attained by fitting the experimental data to the Langmuir and Freundlich isotherm models is presented in Fig. S4. As can be seen, better experimental data fitting was obtained for the Langmuir isotherm model. It seems that in the tested system, the monolayer coverage is more advantageous than the multiple adsorption process. Thus, the adsorption is of chemisorption nature, which involves the formation of coordination bonds between the Cr(VI) ions and S atoms present on the surface of MoS_2_-rGO. The calculated *n* value from the Freundlich isotherm model points out the dominant role of the adsorption process [31]. In Table S1, the adsorption capacities of MoS_2_-based nanomaterials used for the adsorption of Cr(VI) from the aqueous samples are presented. It can be noted that the MoS_2_-rGO exhibits the highest affinity to Cr(VI) ions. The q_max_ value is nearly 6.5 times higher than that reported for the raw MoS_2_.

### Study of potential interferences

The key to practical application of the method is to study the influence of potentially interfering ions and organic matter present in surface water on the Cr(VI) ions recovery. The natural organic matter (NOM) of waters originates from plants and animal decomposition products. Typically, NOM concentration in surface water cover the 0.1–20 mg L^−1^ range. NOM consists of non-humic substances easily decomposed by microorganism and humic substances (HS) more stable than their forerunner. HS including fulvic acids, humic acids, and humins vary in a molecular weight, structure, composition, and positions of functional groups. Despite the differences, all contain carboxyl, phenol, hydroxyl, amine, and quinine groups [32]. In order to study the influence of NOM on the Cr(VI) adsorption, humic acid (HA) and tannic acid (TA) were used as model compounds.

Sample solutions containing 20 ng mL^−1^ of Cr(VI) and various amounts of hypothetically interfering species were prepared, and the preconcentration procedure was performed. If the scope of changes in the recovery was within the range *R* ± 5%, it was assumed that the tested ion does not affect the results. The achieved results are summarized in Table S2.

The conducted studies reveal that the most typical anions (Cl^−^, SO_4_^2−^, NO_3_^−^, CO_3_^2−^, HCO_3_^−^, PO_4_^3−^, HPO_4_^2−^ Br^−^, B_4_O_7_^2−^) and cations (Na^+^, K^+^, Ca^2+^, Mg^2+^, Al^3+^) do not influence the adsorption of Cr(VI) on the MoS_2_-rGO surface. The transition metals do not affect the chromium determination at amounts lower or equal 500-fold excess over Cr(VI) ions. In the case of cations, it results mainly from the electrostatic repulsion between two positively charged specimens. In a view of the soft and hard acids and base theory, sulfur as soft base weakly interacts with alkali and alkaline earth cations which are considered to be hard acids. The resistance to high concentrations of the anions can be partly explained by weaker interaction with positively charged adsorbent surface unlike oxoions of Cr(VI). The presence of humic and tannic acids does not affect the chromium recovery at amounts lower or equal to 250-fold excess over Cr(VI) ions. The conducted studies demonstrated that the method can be applied in the analysis of water samples, even high salinity ones.

### Analytical characteristics

To characterize the described method for hexavalent chromium determination, some parameters, namely (i) linearity range, (ii) detection and quantification limits, (iii) precision at two concentrations of Cr(VI) ions, (iv) enrichment factor, and (v) recovery, were determined. The proportional relationship between the fluorescent radiation intensity and the concentration of the analyte ions was achieved for the concentration range of 1–200 ng mL^−1^ (*R*^2^ = 0.998).

Based on the following equation: LOD = (3/k)(B/t)^1/2^, where k is the count sensitivity (s^−1^ μg^−1^), B is the count rate of blank sample (counts s^−1^), and t is the counting time (s), the LOD value was 0.050 ng mL^−1^. The limit of quantification (LOQ) was computed by multiplying LOD by 3.3 and equaled 0.165 ng mL^−1^. The LOD and LOQ were nearly 1000-fold lower than those for the direct EDXRF analysis of water samples (with the counting time in the 3–30 min range using 100–1000 mL in volume samples). The LOD and LOQ values are much lower than the acceptable concentration of Cr (50 ng mL^−1^) for drinking water suggested by the World Health Organization (WHO) [1]. The precision of the method determined for six replicates at two concentrations, namely 5 ng mL^−1^ and 50 ng mL^−1^, were 3.5 and 1.8%, respectively. The enrichment factor (EF) was calculated as ratio of sensitivity of the DMSPE–EDXRF procedure to the sensitivity of the direct EDXRF measurement. The EF of 3350 is much higher than the typical EF values of FAAS or ICP-OES procedures. It can be explained by the absence of the elution of the analyte from the adsorbent surface. Recovery value of 97 ± 3 for six replicates and chromium concentration of 20 ng mL^−1^ was calculated from the following formula: *R* = ((c_initial_-c_DMSPE_)/c_initial_) × 100%, where *R* is recovery, c_initial_ is concentration of Cr(VI) added to the solution (μg L^−1^), c_DMSPE_ is the concentration of Cr(VI) determined after DMSPE procedure (μg L^−1^).

Comparison of the method for hexavalent chromium determination with the literature data is shown in Table 1. Although FAAS technique is usually considered as favorable due to low costs, availability, operational equipment, and high sample throughput, the LOD for chromium are moderate even after preconcentration step [14, 33]. The best LOD is achieved by combination of microcolumn SPE with ICP-MS. Nevertheless, the high operating costs associated with Ar consumption and operating complexity make it expensive in routine analysis [13]. Due to the fact that both techniques operate on liquid samples analyses, elution step prior to measurements is necessary. This not only extends sample pretreatment time but also may be a source of errors related to analyte loss and sample contamination. Different techniques associated mainly with solid samples have also been used for chromium determination, e.g., TXRF [11], wavelength dispersive X-ray fluorescence spectrometry (WDXRF) [12], and EDXRF [15]. It seems that the preconcentration step based on DSPE with nanosized materials applied as adsorbents significantly broadens their practical application to liquid samples. The very small size of such nanomaterials, as well as the use of small amounts of adsorbents (up to 1 mg) guaranteeing thin samples, simplifies quantitative analysis. In that case, particle size effects and matrix effects can be neglected. XRF analysis of hexavalent chromium was preceded by DSPE using MWCNTs modified with Aliquat 336 [11], thin film of Aliquat 336 supported on GO [12], and GO modified with 3-(2-aminoethylamino) propyltrimethoxysilane (GO-1N) [15]. The LOD range from 0.17 to 3 ng mL^−1^ and they are from 3.4 times to 60 times worse than those for MoS_2_-rGO. The lack of gas consumption for EDXRF measurements and the elution step elimination are the main benefits, when compared with approaches based on the combination of DSPE with ICP-MS or FAAS.

**Table 1:** Comparison of the procedure for hexavalent chromium determination with the existing methods

Preconcentration procedure	Adsorbent	pH	Linearity range, ng mL^−1^	LOD, ng mL^−1^	Detection technique	Matrix	Ref.
DMSPE	MWCNTs modified with Aliquat 336	2 7.5	10–3000 10–500	3 2	TXRF	Water	11
DSPE	Thin film of Aliquat 336-GO	8	n/a	0.35	WDXRF	Red wine	12
Microcolumn SPE	AAPTS-MWCNTs	2.2	0.1–100	0.04	ICP-MS	Water	13
Dispersive MSPE	mf-GO	2	1.0–100	1.4	FAAS	n/a	14
DSPE	GO-1 N	3.5	2–1400	0.17	EDXRF	Water	15
DSPE	TRG-SiO_2_-APTES	1.7	n/a	0.4	UV-VIS	Water	34
DSPE	Fe_3_O_4_@INPs	3	n/a	0.29	FAAS	Water	33
DSPE	Fe_3_O_4_@GO modified with TETA	2	n/a	1.4	FAAS	Water	14
DMSPE	MoS_2_-rGO	2	1–200	0.05	EDXRF	Water	This study

*MWCNTs/IL*, multiwalled carbon nanotubes modified with 1-butyl-3-methyl imidazolium chloride; *AAPTS-MWCNTs*, multiwalled carbon nanotubes modified with 3-(2-aminoethylamino) propyltrimethoxysilane; *mf-GO*, graphene oxide decorated with triethylenetetramine-modified magnetite; *GO-1N*, graphene oxide modified with 3-(2-aminoethylamino) propyltrimethoxysilane; *TRG-SiO*_*2*_*-APTES*, thermally reduced graphene (TRG) modified SiO_2_; *Fe*_*3*_*O*_*4*_*@INPs*, magnetic Cr(VI)-imprinted nanoparticles; *Fe*_*3*_*O*_*4*_*@GO modified with TETA*, graphene oxide decorated with triethylenetetramine-modified magnetite; *n/a*, data not available

### Regeneration of MoS_2_-rGO

From the environmental and economic point of view, MoS_2_-rGO regenerative study is of a key importance. The efficiency of the desorption process was carried out using 2 mL of sodium hydroxide and ammonium hydroxide at a concentration of 1 mol L^−1^. Such eluents were chosen because in the alkaline solution, the deprotonation of surface functional groups is causing an increase in the negative charge on the surface of MoS_2_-rGO and thus increases the desorption of Cr(VI). The desorption percentage obtained for NaOH is 98%, while for NH_3_aq, it does not exceed 80%. Further experiments were carried out using sodium hydroxide as the eluent. The influence of MoS_2_-rGO surface regeneration cycles on the adsorption of Cr(VI) ions was repeated 5 times. The results presented in Fig. S5 show that as the number of cycles increases, the desorption percentage slowly decreases. For the first two adsorption-desorption cycles, no significant decrease in adsorption properties was observed. After 5 cycles, adsorption decreases to 85%. It can be partially ascribed to the adsorbent loss during the regeneration process. It should be mentioned here that a single experiment was carried out using only 1 mg of the adsorbent. The studies show the potential of MoS_2_-rGO as an efficient and recyclable nanomaterial for Cr (VI) adsorption from aqueous samples.

### Determination of chromium species in waters

To demonstrate usefulness of the MoS_2_-rGO to chromium speciation in waters, a sample batch spiked with a known concentration of target analyte ions was prepared. The experiments were conducted according to the “Ultrasound-assisted dispersive micro-solid phase extraction for Cr(VI)” and “Chromium speciation” sections. Cr(III) concentration was computed from the difference between the total amount of Cr(VI) (after oxidation of Cr(III) to Cr(VI) with potassium permanganate) and initial content of Cr(VI) ions. The results listed in Table 2 show the potential of the described method to speciation analysis of chromium at trace and ultratrace levels.

**Table 2:** Chromium speciation in spiked waters (sample volume 50 mL, adsorbent mass 1 mg, sample pH 2, sonication time 10 min, uncertainties correspond to one standard deviation, *n* = 3)

Sample	Added, ng mL^−1^	Found, ng mL^−1^			Recovery, %	
	Cr(VI)	Cr(III)	Cr(VI)	Cr(III)	Cr(VI)	Cr(III)
River water	0	0	< LOD	< LOD	-	-
	0	10	< LOD	9.2 ± 0.5	-	92
	10	0	9.8 ± 0.4	< LOD	98	-
	10	10	9.7 ± 0.5	9.4 ± 0.5	97	94
	10	20	9.8 ± 0.6	19.2 ± 0.7	98	96
	20	10	19.6 ± 0.7	9.4 ± 0.4	98	94
	0	20	< LOD	19.2 ± 0.6	-	96
	20	0	19.8 ± 0.6	< LOD	99	-
Lake water	0	0	< LOD	< LOD	-	-
	0	10	< LOD	9.4 ± 0.5	-	94
	10	0	9.7 ± 0.5	< LOD	97	-
	10	10	9.9 ± 0.6	9.5 ± 0.4	99	95
	10	20	9.7 ± 0.4	19.2 ± 0.8	97	96
	20	10	19.7 ± 0.6	9.3 ± 0.5	98	94
	0	20	< LOD	19.5 ± 0.7	-	96
	20	0	19.6 ± 0.6	< LOD	98	-
Artificial sea water	0	10	< LOD	9.2 ± 0.5	-	92
	10	0	9.6 ± 0.4	< LOD	96	-
	10	10	9.8 ± 0.6	9.3 ± 0.4	98	93
	10	20	9.7 ± 0.4	19.4 ± 0.8	97	97
	20	10	19.7 ± 0.8	9.5 ± 0.4	98	95
	0	20	< LOD	19.4 ± 0.7	-	97
	20	0	19.7 ± 0.8	< LOD	98	-

In order to check the accuracy of the method, analyses of two certified materials, e.g., chromium VI in drinking water (QC1453) and chromium VI in sea water (QC3015), were also carried out. The results, presented in Table 3, are consistent with the certified values, which proves the utility of the method.

**Table 3:** Determination of Cr(VI) in certified materials: chromium VI in drinking water (QC1453) and chromium VI in sea water (QC3015) (sample volume 50 mL, adsorbent mass 1 mg, sample pH 2, sonication time 10 min, uncertainties correspond to one standard deviation, *n* = 3)

CRM	Certified concentration, μg L^−1^	Determined concentration, μg L^−1^	Relative difference, %
QC1453	18.54 ± 1.04	18.2 ± 0.5	− 1.7
QC3015	450 ± 13.9	436 ± 7.5	− 3.3

## Conclusions

Reduced graphene oxide decorated with MoS_2_ was applied for the selective preconcentration of Cr(VI) ions from aqueous samples in the acidic media. The strong affinity of a target analyte to the MoS_2_-rGO nanoparticles results from both electrostatic interaction and outsphere surface complexation. The adsorption capacity of 583.5 mg g^−1^ is almost 6.5 times higher than that reported for the raw MoS_2_. A better description of experimental data to the Langmuir isotherm model suggests that in the studied system, chemisorption occurs. The method can be used not only for a sensitive determination of hexavalent chromium but also for chromium speciation. The conducted studies have demonstrated a great potential of the method to water sample analyses even in the case of high salinity ones. Choosing EDXRF as the measurement technique, as an alternative to commonly applied ICP-OES, ICP-MS, or FAAS, eliminates the necessity of elution step which minimizes the risk of the analyte loss or contamination of a sample. Unlike the more generally used spectroscopic techniques, EDXRF does not require gases for carrying out the measurements, which makes the method less expensive and environmental friendly. Although the detection limits offered by the EDXRF technique are not sufficient for trace or ultratrace analyses, the DMSPE/EDXRF procedure being the scope of the work combines the essential advantages of the abovementioned procedures, which broadens the scope of its possible applications.

## Electronic supplementary material

ESM 1 (DOCX 1799 kb)
