# Supplementary material for: Ultrasound-assisted dispersive micro-solid phase extraction using molybdenum disulfide supported on reduced graphene oxide for energy dispersive X-ray fluorescence spectrometric determination of chromium species in water
Source: Mikrochim Acta. 2020 Sep 2;187(9):542. doi: 10.1007/s00604-020-04476-4 (PMC7467914; doi:10.1007/s00604-020-04476-4)
Supplement: Supplementary file 1 — (DOCX 1799 kb) [file 604_2020_4476_MOESM1_ESM.docx]

**Electronic Supplementary Material**

**on the Microchimica Acta publication entitled**

**Ultrasound-assisted dispersive micro-solid phase extraction using molybdenum disulfide supported on reduced graphene oxide for energy dispersive X-ray fluorescence spectrometric determination of chromium species in water**

**Katarzyna Pytlakowska,^1^ Karina Kocot,^1^ Michał Pilch^2^, Maciej Zubko^3^**

**^1^Institute of Chemistry, University of Silesia, Szkolna 9, 40-006 Katowice, Poland**

**^2^Institute of Physics, University of Silesia, 75 Pułku Piechoty 1a, 41-500 Chorzów, Poland**

**^3^Institute of Materials Science, University of Silesia, 75 Pułku Piechoty 1a, 41-500 Chorzów, Poland**

**Influence of sample pH on adsorption of Cr(VI) ions on MoS_2_-rGO surface**

Adsorption of Cr(VI) ions on the MoS_2_-rGO strongly depends on a sample pH. It influences both the charge of nanoadsorbent surface and ionic forms of a target analyte present in the aqueous sample. The distribution diagram of Cr(VI) species at different sample pH values depicted in Fig. S1, shows that its anionic forms are present in the aqueous media in the whole studied pH range. At acidic conditions HCrO_4_^-^ ions dominate while in basic solutions Cr(VI) occurs in the form of CrO_4_^2-^ ions. Therefore, the charge of MoS_2_-rGO surface plays a crucial role in the adsorption mechanism. In order to select the optimum pH conditions for the preconcentration of hexavalent chromium ions, a batch of samples was prepared. Samples of 50 mL volume contained constant amount of adsorbent, and the same concentration of examines anions were sonicated for 10 min. The effect of sample pH on the adsorption process was examined in 1-10 pH range for the following species: Cr(VI), Cr(III), As(III), As(V), Se(IV), and Se(VI). The results are shown in Fig. S1. As can be seen in acidic media adsorption of chromium species on MoS_2_-rGO surface is selective. The highest extraction efficiency is observed at pH 2. This phenomena can be assigned to the protonation of MoS_2_-rGO surface in the acidic environment, resulting in a positive charge surface, which enables electrostatic interaction with anionic chromium species. Further increase of sample pH results in the decrease of the adsorption up to pH 5. Such behavior can be explained by the reduction of positive surface charge on the adsorbent nanosheets and the repulsion between two negatively charged specimens. On the other hand, there is also a possibility of the reduction of Cr(VI) species to Cr(III) promoted in the acidic media according to the equation: HCrO_4_^-^+7H^+^+ 3e⮀Cr^3+^+4H_2_O. However, in neutral media, where adsorbent surface is negatively charged, the adsorption slightly increases, which can be explained by the presence of Cr(III) ions present in the solution. The remained studied anions are not quantitatively adsorbed within the studied pH range.

It should be pointed here, that in a view of the soft and hard acids and bases theory sulfur plays as soft base while Cr(VI) ions are considered to be a hard acid. Although the interaction between Cr(VI) and S atoms is weak, outsphere surface complexation is possible and such interaction was confirmed in literature data [2]. Nevertheless, in the adsorption process of Cr(VI) ions on MoS_2_-rGO electrostatic interaction between negative chromium ions and positively charged surface dominates.


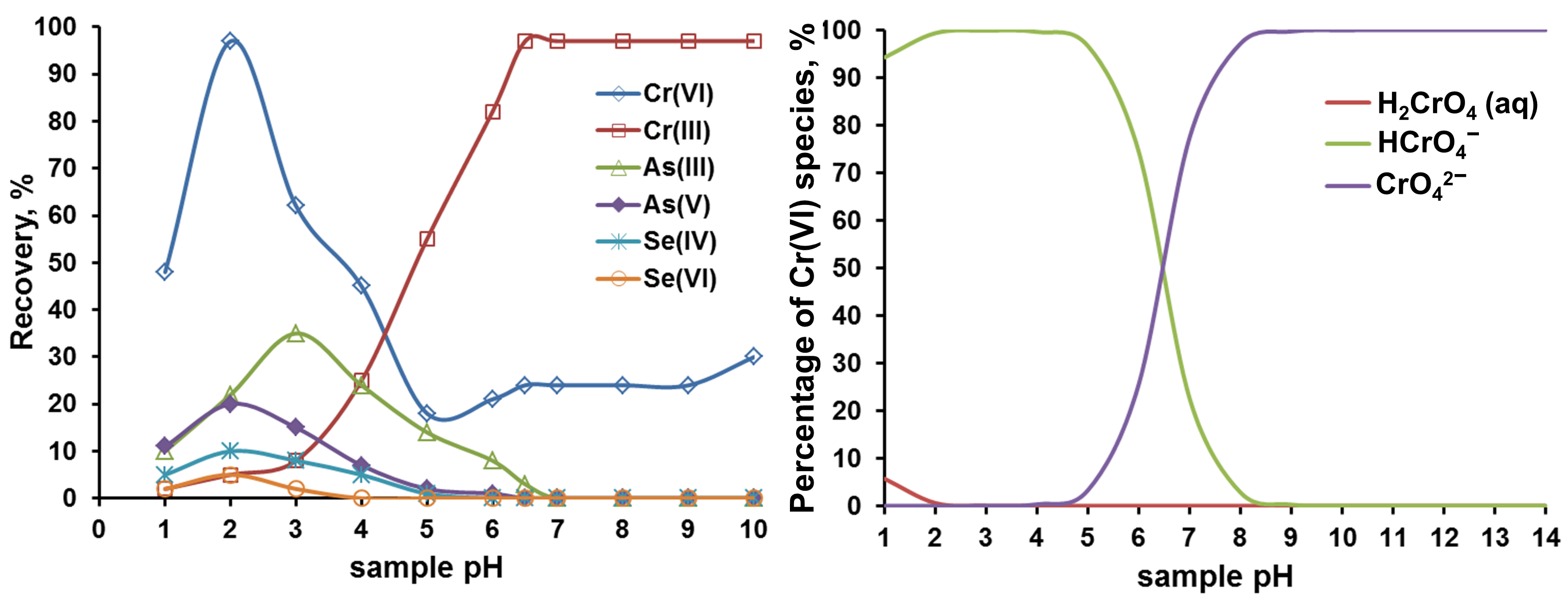


**Fig. S1**. (a) Influence of pH (sample volume 50 mL, metal ions concentration 100 ng mL^-1^, adsorbent mass 1 mg, sonication time 10 min, n=3) on the extraction of Cr(III), Cr(VI), As(III), As(V), Se(IV) and Se(VI) ions; (b) speciation distribution diagram for Cr(VI) species in relation to sample pH [1].

**Influence of MoS_2_-rGO mass on the extraction of Cr(VI) ions**

Adsorbent mass influences on both the adsorption effectiveness and further EDXRF measurements. Generally, an increase in adsorption efficiency is observed with the increase in adsorbent mass. On the other hand, from the EDXRF point of view, the most preferred samples are those in the form of thin solid targets, for which matrix correction can be skipped [3]. Thus, when optimizing the adsorbent mass, a trade-off between adsorption efficiency and sample thickness should be achieved. The influence of MoS_2_-rGO mass on the Cr(VI) recovery was investigated in the range of 0.8–1.5 mg. As can be seen in Figure S2, with the increase of MoS_2_-rGO mass up to 1 mg an increase in adsorption efficiency is observed. Further increase in the adsorbent mass has no significant effect on the recovery of Cr(VI) ions. Subsequent studies were performed using 1 mg of MoS_2_-rGO.


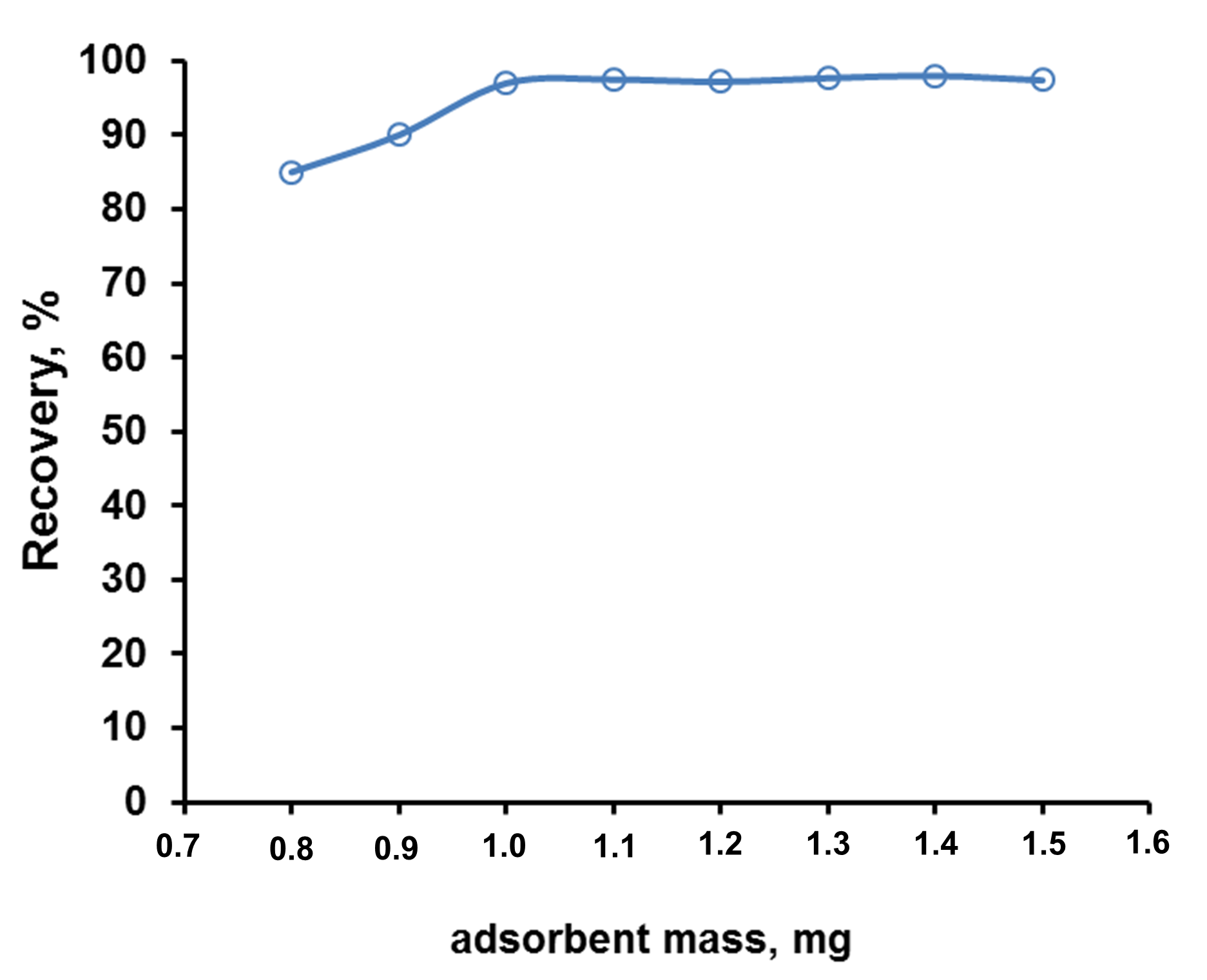


**Fig. S2.** Influence of adsorbent mass on extraction efficiency, given as recovery percentage, of Cr(VI) ions (sample volume 50 mL, Cr(VI) concentration 100 ng mL^-1^, sample pH=2, sonication time 10 min, n=3).

**Influence of sample volume and contact time on the extraction of Cr(VI) ions**

The contact time between analyte and adsorbent is closely related to the volume of the sample. It is well-known that the larger volume of the sample is, the longer time is necessary for the establishment of the adsorption equilibrium. The sample volume affects also the time of sample filtration. An additional factor related to both parameters is the degree of the adsorbent dispersion in the solution as well as analytes diffusion. Thus, in the dispersive solid-phase extraction the preconcentration step is most commonly accelerated by mechanical agitation or ultrasonic waves. Therefore, the effect of contact time and sample volume was optimized simultaneously. To obtain the most effective mass transfer between two phases, ultrasounds and stirring were employed. The effect of sample volume and contact time were optimized in 25-100 mL and 0-120 min ranges, respectively.

As is illustrated in Fig. S3, the time required to achieve equilibrium state of the adsorption process is nearly 5 times shorter in the case of ultrasounds than for mechanical agitation. For 25 mL-in volume samples the highest adsorption of Cr(VI) ions on MoS_2_-rGO surface was obtained after 5 and 30 min using appropriate ultra-sounds assisted and stirring aided DMSPE. With the increase of sample volume, the contact time was lengthened and for samples with a volume of 100 mL it was 20 and 90 min, respectively. Considering abovementioned parameters together with the filtration time, in the course of further tests, the ultrasound assisted extraction of Cr (VI) ions from 50 mL samples was chosen, for which the analyte contact time with the adsorbent surface was 10 minutes.


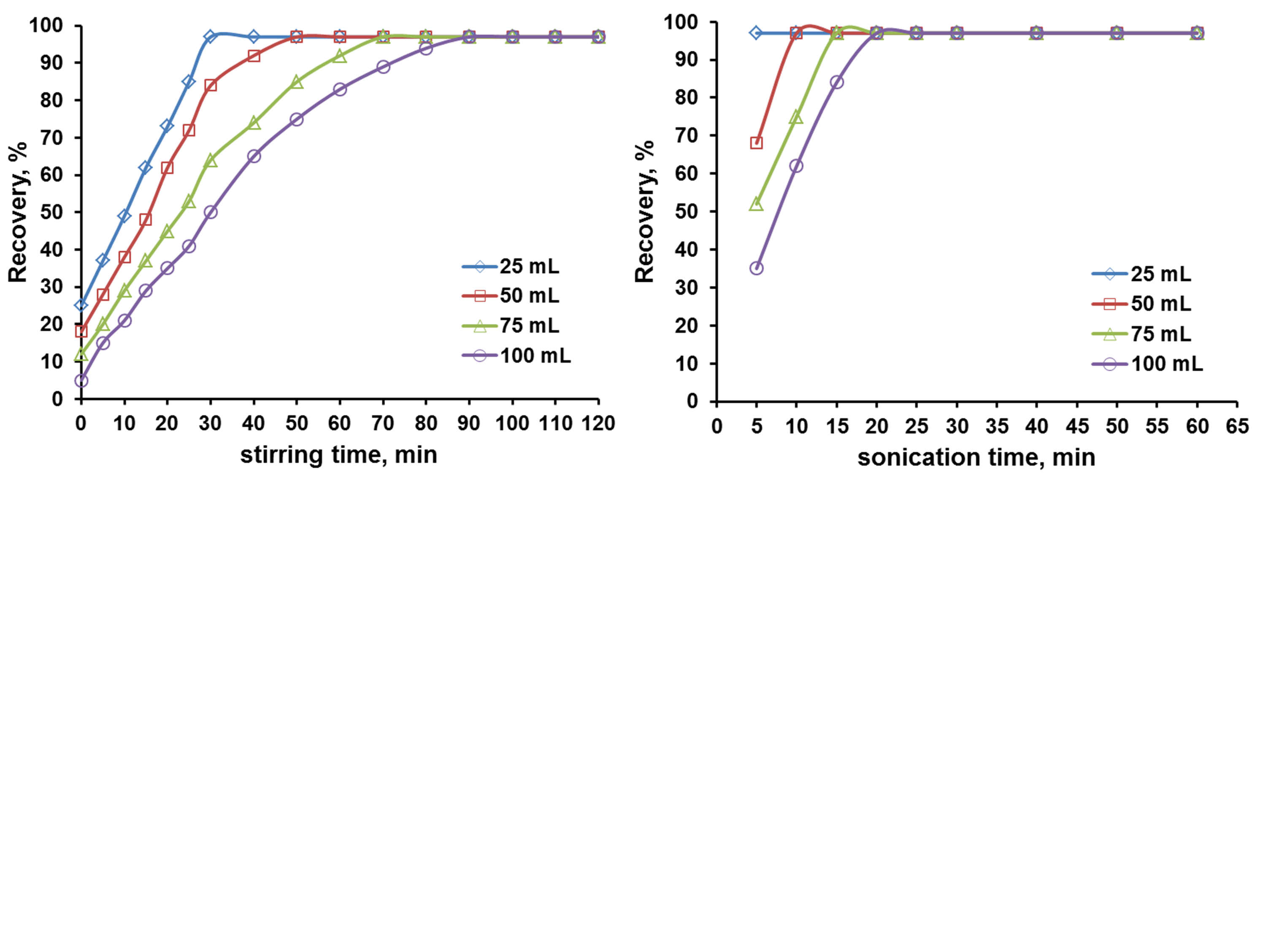


**Fig. S3**. Influence of (a) stirring time or (b) sonication time and sample volume on extraction efficiency, given as recovery percentage, of Cr(VI) ions (sample pH=2, adsorbent mass 1 mg, Cr(VI) concentration 100 ng mL^-1^, n=3).


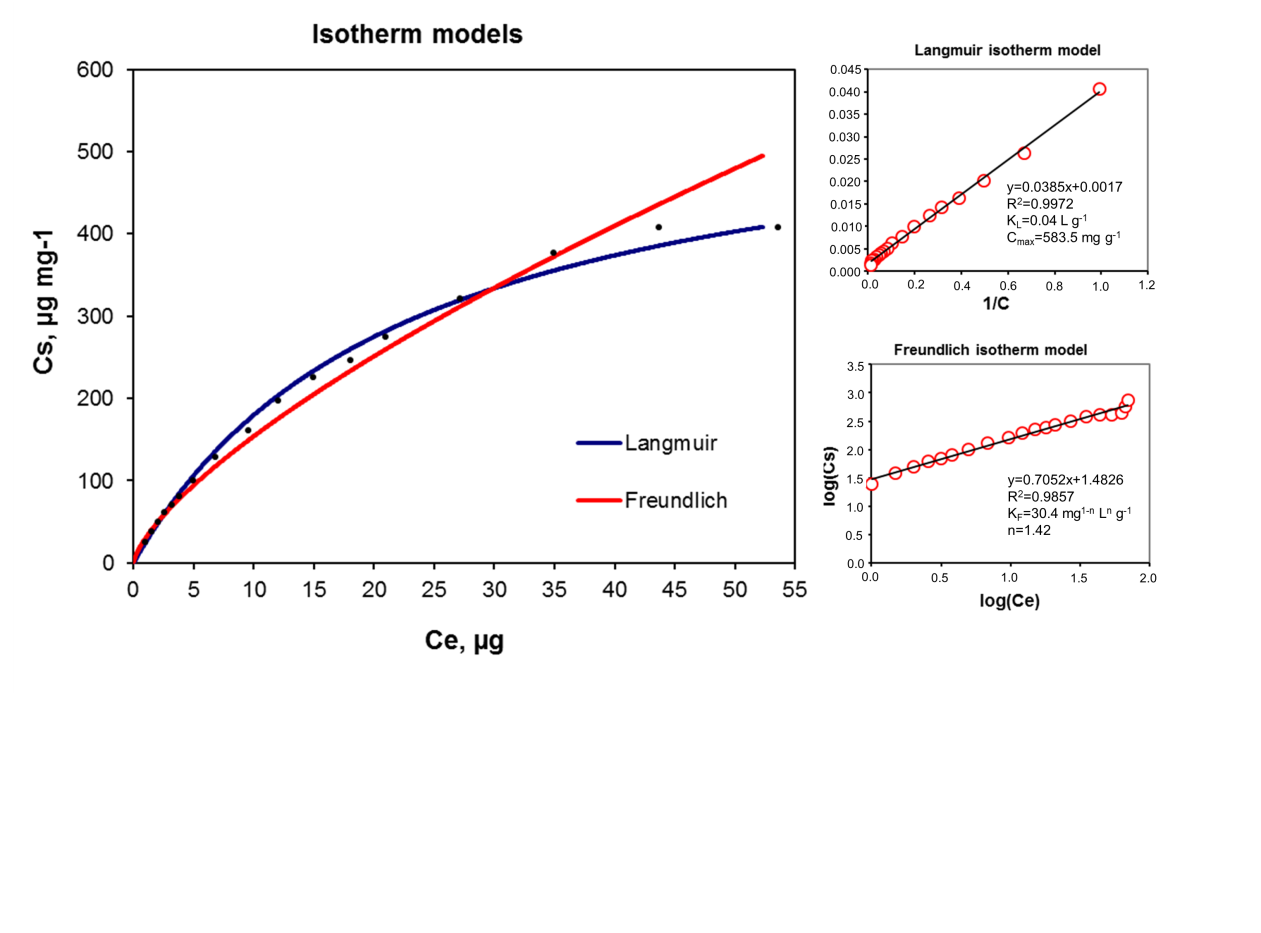


**Fig. S4**. The Langmuir and Freundlich adsorption isotherms for Cr(VI) ions (sample pH=2, adsorbent mass 1 mg, T=20 ºC, sample volume 25 mL, stirring time 180 min).

**Table S1**. Comparison of the adsorption capacity of MoS_2_-based nanomaterials with respect to hexavalent chromium

| Adsorbent | pH | q_max_, mg g^-1^ | Ref. |
| --- | --- | --- | --- |
| MoS_2_ | 6 | 84.03 | 4 |
| MoS_4_-LDH | ca. 7.6 | 130 | 5 |
| MoS_2_@Fe_3_O_4_ | 5 | 218.18 | 6 |
| SDS-MoS_2_ |  | 63.92 | 7 |
| LDHs@MoS_2_ | 5 | 76.3 | 2 |
| PVP/MoS_2_  PAM/MoS_2_ | 5  5 | 142.2  84.9 | 8 |
| MoS_2_/rGO | 2  4.6 | 268.82  192.63 | 9 |
| MoS_2_-rGO | 2 | 583.5 | this study |

MoS_2_-molybdenum disulfide; MoS_4_-LDH-Mg/Al layered double hydroxide (Mg/Al-LDH) intercalated with MoS_4_^2-^, MoS_2_@Fe_3_O_4_-magnetic nanoparticles (Fe_3_O_4_NPs) decorated with MoS_2_, SDS-MoS_2_-sodium dodecyl [sulfate](https://www.sciencedirect.com/topics/earth-and-planetary-sciences/sulfate) intercalated molybdenum disulfide; LDHs@MoS_2_- molybdenum disulfide coated Mg/Al layered double hydroxide composite, PVP/MoS_2_-polyvinylpyrrolidone intercalated molybdenum disulfide composite, PAM/MoS_2_-polyacrylamide intercalated molybdenum disulfide composite, MoS_2_/rGO-reduced graphene oxide decorated with molybdenum disulfide

**Table S2**. Tolerance limits of potentially interfering ions (sample pH=2, concentration of Cr(VI) 20 ng mL^-1^, sample volume 50 mL, sonication time 10 min, uncertainties correspond to one standard deviation, n=3).

| Interference species | Added as | Analyte to potentially interfering ion ratio | Recovery, % |  |
| --- | --- | --- | --- | --- |
| Cl^-^ | NaCl | 1:200000 | 96±3 |  |
| Na^+^ | NaCl | 1:100000 | 96±3 |  |
| SO_4_^2-^ | Na_2_SO_4_ | 1:25000 | 96±2 |  |
| Mg^2+^ | MgCl_2_·6H_2_O | 1:12000 | 95±4 | |
| NO_3_^-^ | KNO_3_ | 1:10000 | 95±4 | |
| Ca^2+^ | CaCl_2_·2H_2_O | 1:5000 | 95±3 | |
| K^+^ | KCl | 1:5000 | 97±4 | |
| Al^3+^ | Al_2_(SO_4_)_3_ | 1:5000 | 96±3 | |
| CO_3_^2-^ | Na_2_CO_3_ | 1:2500 | 95±4 | |
| HCO_3_^-^ | NaHCO_3_ | 1:2500 | 97±3 | |
| PO_4_^3-^ | Na_3_PO_4_ | 1:2000 | 98±4 | |
| HPO_4_^2-^ | Na_2_HPO_4_ | 1:2000 | 96±3 | |
| Br^-^ | KBr | 1:2000 | 95±3 | |
| B_4_O_7_^2-^ | Na_2_B_4_O_7_·10H_2_O | 1:2000 | 97±3 | |
| Mn^2+^ | MnCl_2_ | 1:500 | 96±3 | |
| Ni^2+^ | Ni(NO_3_)_2_ | 1:500 | 94±3 | |
| Co^2+^ | Co(NO_3_)_2_ | 1:500 | 95±2 | |
| Fe^3+^ | FeCl_3_ | 1:500 | 94±2 | |
| As^3+^ | Na_3_AsO_3_ | 1:500 | 99±4 | |
| HA | Humic acid | 1:250 | 102±3 | |
| TA | Tannic acid | 1:250 | 101±5 | |

**
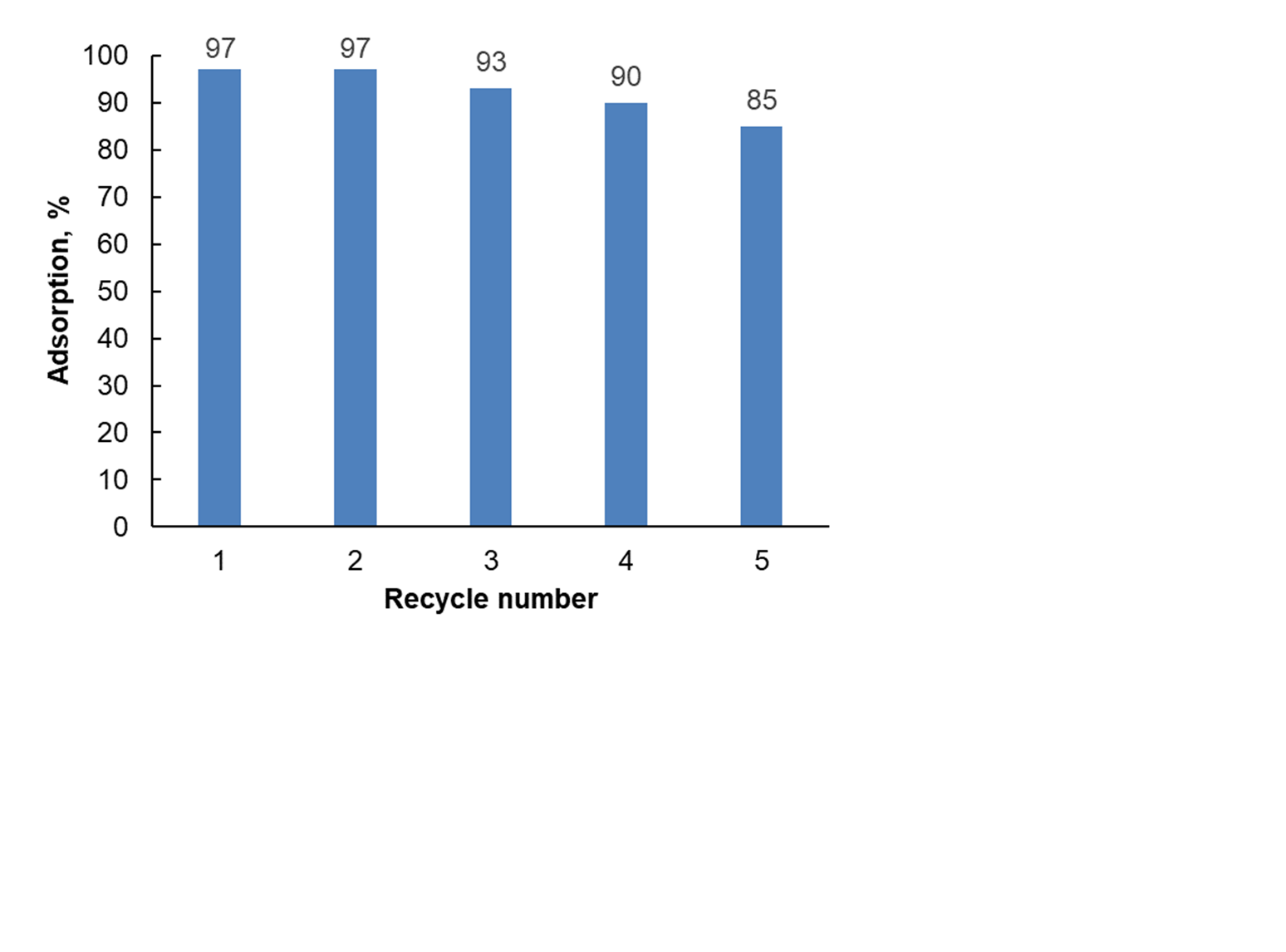
**

**Fig. S5.** Adsorption of Cr(VI) on the MoS_2_-rGO nanosheets at different regeneration cycles (concentration of Cr(VI) 100 ng mL^-1^, adsorbent mass 1 mg, sample pH=2, sonication time 10 min, sample volume 50 mL, n=3).

**References**

1. MINTEQA2 Equilibrium Speciation Model (2006) https://www.epa.gov/ceam/minteqa2-equilibrium-speciation-model. Accessed 08.09.19
2. Wang J, Wang P, Wang H, Dong J, Chen W, Wang X, Wang S, Hayat T, Alsaedi A, Wang X (2017) Preparation of molybdenum disulfide coated Mg/Al layered double hydroxide composites for efficient removal of chromium (VI). ACS Sustainable Chem Eng 5:7165–7174. https://doi.org/10.1021/acssuschemeng.7b01347
3. R. Sitko R (2009) Quantitative X-ray fluorescence analysis of samples of less than 'infinite thickness': Difficulties and possibilities. Spectrochim Acta B 64:1161−1172. https://doi.org/10.1016/j.sab.2009.09.005
4. Sun H, Wu T, Zhang Y, Ng DHL, Wang G (2018) Structure-enhanced removal of Cr(VI) in aqueous solutions using MoS_2_ ultrathin nanosheets. **New J Chem** **42**:9006–9015. https://doi.org/10.1039/C8NJ01062E
5. Ma L, Islam SM, Liu H, Zhao J, Sun G, Li H, Ma S, Kanatzidis MG (2017) Selective and efficient removal of toxic oxoanions of As(III), As(V), and Cr(VI) by layered double hydroxide intercalated with MoS_4_^2–^. Chem Mater 29:3274−3284. https://doi.org/10.1021/acs.chemmater.7b00618
6. Kumar ASK, Jiang S-J, Warchoł JK (2017) Synthesis and characterization of two-dimensional transition metal dichalcogenide magnetic MoS_2_@Fe_3_O_4_ nanoparticles for adsorption of Cr(VI)/Cr(III). ACS Omega 2:6187−6200. https://doi.org/10.1021/acsomega.7b00757
7. Wang J, Zhang R, Huo Y, Ai Y, Gu P, Wang X, Li Q, Yu S, Chen Y, Yu Z, Chen J, Wang X (2019) Efficient elimination of Cr(VI) from aqueous solutions using sodium dodecyl sulfate intercalated molybdenum disulfide. Ecotox Environ Safe 175:251–262. https://doi.org/10.1016/j.ecoenv.2019.03.064
8. Wang J, Wang X, Zhao G, Song G, Chen D, Chen H, Xie J, Hayat T, Alsaedi A, Wang X (2018) Polyvinylpyrrolidone and polyacrylamide intercalated molybdenum disulfide as adsorbents for enhanced removal of chromium(VI) from aqueous solutions. Chem Eng J [334](https://www.sciencedirect.com/science/journal/13858947/334/supp/C):569–578. https://doi.org/10.1016/j.cej.2017.10.068
9. Jiang X, Luo H, Yin Y, Zhou W (2017) Facile synthesis of MoS_2_/reduced graphene oxide composites for efficient removal of Cr(VI) from aqueous solutions. RSC Adv 7:24149–24156. https://doi.org/10.1039/C7RA03531D
